# Assessment of two complementary influenza surveillance systems: sentinel primary care influenza-like illness versus severe hospitalized laboratory-confirmed influenza using the moving epidemic method

**DOI:** 10.1186/s12889-019-7414-9

**Published:** 2019-08-13

**Authors:** Núria Torner, Luca Basile, Ana Martínez, Cristina Rius, Pere Godoy, Mireia Jané, Ángela Domínguez, The Working Group on PIDIRAC Sentinel Surveillance of Catalonia, J. Aizpurua, J. Alonso, J. Azemar, P. Aizpurua, P. M. Ardaya, M. D. Basas, J. Batalla, P. Biendicho, M. Bonet, M. Callado, S. Campos, J. M. Casanovas, E. Ciurana, M. Clapes, J. M. Cots, D. De la Rica, I. Domingo, G. Elizalde, P. Escapa, S. Fajardo, E. Fau, O. Fernandez, M. Fernandez, C. Ferrer, A. Forcada, E. Fos, G. Gadea, J. Garcia, R. Garcia, C. Gatius, M. J. Gelado, M. Grau, M. Grivé, M. C. Guzman, R. Hernández, G. Jimenez, A. Juscafresa, A. M. LLussa, C. López, L. Kristensen, E. Macià, A. Mainou, E. Marco, M. Martínez, J. G. Martínez, K. V. Marulanda, R. Masa, X. Moncosí, M. A. Naranjo, D. Navarro, E. Ortolà, F. París, M. M. Pérez, C. Pozo, R. Pujol, A. Ribatallada, G. Ruiz, S. Sabaté, R. Sanchez, N. Sarrà, E. Tarragó, A. M. Teixidó, A. Torres, E. Valén, D. Van Esso, C. Van Tarjcwick, R. Vink Schoenholzer, E. Zabala, M. A. Marcos, M. D. M. Mosquera, P. de Molina, E. Rubio, R. Isanta, A. Anton, T. Pumarola, A. Vilella, P. Gorrindo, E. Espejo, M. Andrés, F. Barcenilla, G. Navarro, I. Barrabeig, J. Pou, P. Alvarez, E. Plasencia, J. Rebull, M. R. Sala, M. Riera, N. Camps, N. Follia, A. Oller, P. Godoy, P. Bach, C. Rius, R. Hernández, R. Perez, R. Torra, M. Carol, S. Minguell, R. Marce, G. Garcia-Pardo, M. Olona, A. Alvarez, J. M. Ramon, J. M. Mòdol, G. Mena, M. Campins, C. Massuet, G. Tora, J. Ferràs, G. Ferrús

**Affiliations:** 1 Department of Health, Public Health Agency of Catalonia, Generalitat of Catalonia, Salvany Building, Roc Boronat 81-95, 08005 Barcelona, Catalonia Spain; 2 CIBER Epidemiología y Salud Pública (CIBERESP) Institute Carlos III, Madrid, Spain; 3 Medicine Department, University of Barcelona, Barcelona, Spain; 4 Public Health Agency of Barcelona, Barcelona, Spain

**Keywords:** Sentinel surveillance, Influenza, Epidemic, Threshold, Primary health care, Influenza like illness, Hospitalization

## Abstract

**Background:**

Monitoring seasonal influenza epidemics is the corner stone to epidemiological surveillance of acute respiratory virus infections worldwide.

This work aims to compare two sentinel surveillance systems within the Daily Acute Respiratory Infection Information System of Catalonia (PIDIRAC), the primary care ILI and Influenza confirmed samples from primary care (PIDIRAC-ILI and PIDIRAC-FLU) and the severe hospitalized laboratory confirmed influenza system (SHLCI), in regard to how they behave in the forecasting of epidemic onset and severity allowing for healthcare preparedness.

**Methods:**

Epidemiological study carried out during seven influenza seasons (2010–2017) in Catalonia, with data from influenza sentinel surveillance of primary care physicians reporting ILI along with laboratory confirmation of influenza from systematic sampling of ILI cases and 12 hospitals that provided data on severe hospitalized cases with laboratory-confirmed influenza (SHLCI-FLU). Epidemic thresholds for ILI and SHLCI-FLU (overall) as well as influenza A (SHLCI-FLUA) and influenza B (SHLCI-FLUB) incidence rates were assessed by the Moving Epidemics Method.

**Results:**

Epidemic thresholds for primary care sentinel surveillance influenza-like illness (PIDIRAC-ILI) incidence rates ranged from 83.65 to 503.92 per 100.000 h. Paired incidence rate curves for SHLCI –FLU / PIDIRAC-ILI and SHLCI–FLUA/ PIDIRAC-FLUA showed best correlation index’ (0.805 and 0.724 respectively). Assessing delay in reaching epidemic level, PIDIRAC-ILI source forecasts an average of 1.6 weeks before the rest of sources paired. Differences are higher when SHLCI cases are paired to PIDIRAC-ILI and PIDIRAC-FLUB although statistical significance was observed only for SHLCI-FLU/PIDIRAC-ILI (*p*-value Wilcoxon test = 0.039).

**Conclusions:**

The combined ILI and confirmed influenza from primary care along with the severe hospitalized laboratory confirmed influenza data from PIDIRAC sentinel surveillance system provides timely and accurate syndromic and virological surveillance of influenza from the community level to hospitalization of severe cases.

## Background

Influenza seasonal epidemics occur yearly during each hemisphere’s winter season, whether it be in the Northern or Southern hemisphere. Acute respiratory infection caused by influenza virus ranges from mild to severe and can even cause death in at risk population such as the elderly and young infants. Annual epidemics are estimated to result in about 3 to 5 million cases of severe illness, and about 250,000 to 500,000 deaths worldwide. Hospitalization and death occur mainly among these high risk groups, which include also pregnant women and anyone with underlying medical conditions such as diabetes, obesity, cardiovascular or chronic obstructive pulmonary disease [1, 2].

Influenza epidemics can cause an overload in the healthcare system because of high incidence rates of affected population, especially during peak illness periods. Influenza surveillance is made up information on morbidity and mortality as well as virological assessment of circulating influenza viruses. The resulting assessment and interpretation of these data can support public health authorities to implement special measure to strengthen capacity and preparedness which will in turn translate into action a reduction of morbidity and mortality. In most developed countries a network of sentinel physicians report cases attended for influenza-like illness (ILI), which is used as a proxy to estimate influenza virus circulation, and on the other hand collect samples for virological confirmation, identification of causative virus and describe predominant circulating type and subtype of influenza virus [3].

In some countries community-based ILI reporting systems and indicators such as school and workplace absenteeism data are recorded weekly and have been used to estimate a proxy influenza intensity in combination with routine outpatient physician consultations and hospital influenza sentinel surveillance data [4, 5].

Sentinel surveillance networks in primary care are a readily available and basic source of data that estimates disease burden of ILI cases as well as laboratory-confirmation of respiratory viruses, including influenza, by systematic sampling of ILI cases. From these data thresholds can be established to pinpoint the start and termination of influenza seasonal epidemics. Other relevant information gathered, such as patients’ vaccination status, will allow for vaccine effectiveness estimation [6]. Influenza causes high rates of consultations, hospitalization and, in severe cases, death every year and vaccination is the main preventive measure undertaken globally, but despite policy recommendations, influenza vaccination uptake remains suboptimal in most countries. Low levels of vaccination coverage among at high risk groups is a missed opportunity for preventing influenza infection and complications that can derive into hospitalization and severe outcome [6]. In addition, influenza vaccine manufacturing is conditioned to antigenic changes in the circulating strains detected by the surveillance systems and when these changes occur with respect to onset of seasonal epidemics. Furthermore, immune response varies depending on age, underlying diseases, and immunosuppression [7] .

In all, vaccine effectiveness is hampered and derives into low vaccine uptake. These facts underscore the importance of any surveillance data available whatsoever, making them essential for the detection and preparedness for any changes or unusual behavior of seasonal and non-seasonal influenza. However, a limitation to sentinel surveillance is the limited coverage of population within a geographical area and the bias towards young children who are more prone to seek medical assistance. During the 2009 influenza pandemic, the need to assess influenza severity became evident and from then on a number of European such as Spain, as well as other, countries introduced surveillance of severe disease and death due to influenza into their seasonal influenza surveillance systems [8–10].

In Catalonia severe hospitalized confirmed influenza data from sentinel hospitals are informed on a weekly basis to the Daily Acute Respiratory Infection Sentinel Surveillance System of Catalonia (PIDIRAC) [9]. Identification of admissions due to pneumonia, to intensive care units and deaths among hospitalized laboratory confirmed influenza cases is available and Information on hospitalization, clinical presentation, underlying diseases, vaccination and antiviral treatment as well as and mortality is added to notification records for influenza cases.

Hospital admissions for influenza and pneumonia through emergency departments provide some information on community cases, yet it is currently not easy to track a patient’s journey from community care (primary care consultations) into the hospital and the lack of denominator (source population) data is a major limitation of hospital-based surveillance [8].

In October 2010, the Public Health Agency of Catalonia of the Department of Health in Catalonia, a region in the northeast of Spain with 7.5 million inhabitants, implemented the surveillance of severe hospitalized cases of confirmed influenza as a tool to complement information provided by the influenza sentinel system based on primary healthcare physicians implemented since 1998 [9]. The aim of this study is to assess and compare which of the two networks, primary care and hospital based severe cases, grants a more timely description of influenza epidemics.

## Methods

### Data sources

The PIDIRAC primary care network was composed by 60 sentinel physicians, general practitioners and pediatricians, covering 1% of the total population of Catalonia. Representativeness in terms of age, sex, and urban or rural setting was considered. Sentinel physicians reported ILI cases detected in their reference populations on a daily basis, following the European ILI case definition [9, 11]. For virological influenza surveillance, systematic swab sampling (nasal or nasopharyngeal) of the first 2 ILI patients each week which are sent to the network-affiliated laboratory (Hospital Clinic, Barcelona) for detection of a batch of respiratory viruses, including influenza. Samples are confirmed by polymerase chain reaction (PCR) and/or culture of nasopharyngeal swabs. Respiratory tract samples were processed within 24 h of receipt at the laboratory. Subsequently, two specific one-step multiplex real-time PCR was carried out for typing A/B influenza virus and subtyping influenza A virus [12].

The information collected in the PIDIRAC includes data on demographic, clinical and virological characteristics, seasonal vaccination status, chronic conditions, and pregnancy. Indicators provided by the PIDIRAC sentinel system for seven influenza seasons (2010–2011 to 2016–2017) was analyzed in the study. Indicators for this data source were the incidence rate of ILI syndromes (PIDIRAC-ILI) calculated according to weekly population under surveillance by each sentinel physician of the network that is reporting. Laboratory influenza confirmed isolates from reported ILI cases from primary care (PIDIRAC-FLU) were registered as weekly incidence rate positive to influenza. The latter indicator was separated as to influenza virus type A (PIDIRAC-FLUA) and type B (PIDIRAC-FLUB).

The surveillance of Severe Hospitalized Laboratory Confirmed Influenza (SHLCI) was made up by twelve tertiary care hospitals representing a mean of 63.6% of the population under surveillance. Coverage increased from 53.3 to 94% of the total Catalan population in the 2015–2016 season. The catchment population for SHLCI surveillance was the population assigned to each hospital by the health system as a referral facility for any condition leading to hospitalization.

The system is based on the notification of those laboratory confirmed influenza hospitalized cases admitted to the sentinel facility and who met the SHLCI case definition: Any case with clinical features compatible with influenza (sudden onset of symptoms and at least one of the following four systemic symptoms: fever or feverishness,malaise,headache .myalgia and at least one of the following three respiratory symptoms: cough,sore throat, shortness of breath) [11], requiring hospitalization for clinical severity (at least one of the following criteria: pneumonia, septic shock, acute respiratory distress syndrome, multiple organ dysfunction syndrome, or admission to intensive care unit (ICU) [9].

Only cases that were laboratory confirmed for influenza and met the SHLCI case definition were reported, regardless of whether they had been previously admitted to emergency room and transferred to ward or admitted to ward on initial consultation.

Date of hospitalization and type and subtype of influenza virus were collected, other clinical and sociodemographic variables and outcomes, although collected, were not relevant for this study. We used data obtained from the surveillance of SHLCI for the same study period as in primary care surveillance for ILI, to calculate the cumulative hospitalization rates for each season and threshold for epidemic onset.

Indicators for this data source were the incidence rate of severe hospitalized influenza laboratory confirmed cases (SHLCI -FLU) and separated as to influenza virus type A (SHLCI-FLUA) and type B (SHLCI-FLUB). For SHLCI-FLU and SHCLI-FLUA and FLUB the same method was applied to calculate epidemic thresholds using MEM system [13, 14].

### Statistical analysis

Epidemic thresholds for incidence rates by the Mobile Epidemics Method (MEM) were calculated and categorized as to intensity of epidemic (baseline, low, moderate, high and very high) for the PIDIRAC-ILI and PIDIRAC-FLU indicators taking into account the seven epidemic seasons included in the study [14, 15].

Epidemic curves obtained from both data sources were paired as PIDIRAC-ILI vs SHLCI-FLU, PIDIRAC-FLU vs SHLCI-FLU, PIDIRAC-FLUA vs SHLCI-FLUA and PIDIRAC-FLUB vs SHLCI-FLUB. For each pair, normal partial correlation were calculated at log scale. The week at which incidence rate exceeds estimated epidemic threshold level was used as an indicator to identify whether one source forecasts the onset of the epidemic activity with respect to the other. To calculate this factor by pairs the Wilcoxon signed rank test was carried out.

To compare intensity of influenza epidemic observed by both data sources, correlation of intensity was calculated for each pair and the percentage of agreement within each epidemic threshold level. Correlation was defined as the proportion of weeks in which the observed intensity of activity for one source matches with the observed by the other source. To identify whether one source forecasts the onset of the epidemic with respect to the other for each season and for each indicator, the week at which incidence rate exceeds the estimated epidemic threshold level, this factor was studied by pairs according to Wilcoxon signed rank test.

The analysis was performed using the SPSS v.18 statistical package and the R v3.3.0 statistical software (http://cran.r-project.org).

## Results

**Fig. 1:**
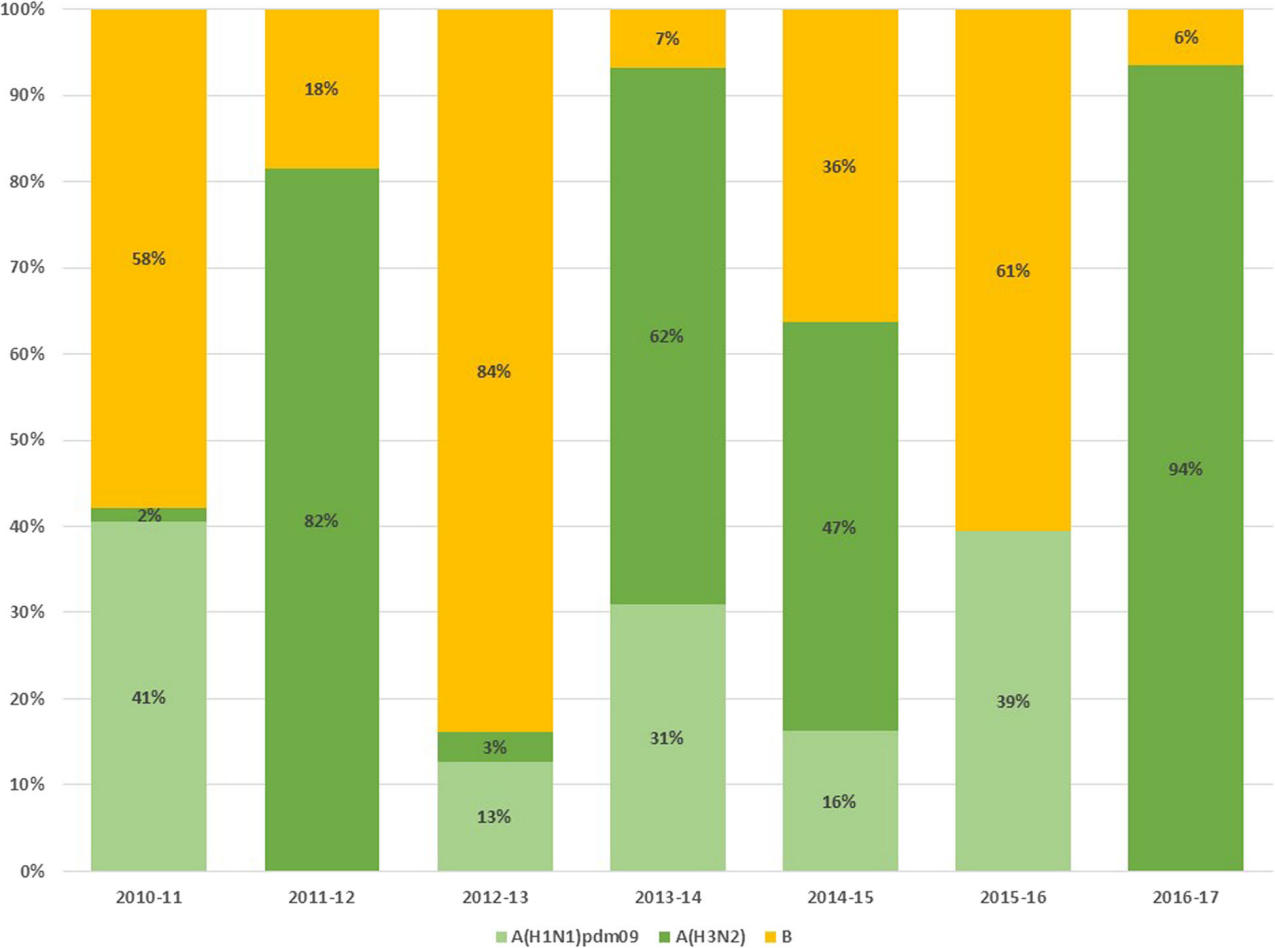
Proportion of circulating influenza virus according to type/subtype A (H1N1) pdm09, A (H3N2) and B per season

Estimated epidemic threshold levels obtained by the MEM method for each source of data are presented in Table 1. Of the seven seasons under study, there was co-circulation of influenza virus type A and B in 3 of seasons (2010–2011, 2014–2015 y 2015–2016), in the 2011–2012 season influenza virus A was predominant and some sporadic circulation of influenza type B virus, in the 2012–2013 season there was predominant circulation of virus B with sporadic cases of A, and in seasons 2013–2014 and 2016–2017 there was absolute predominance of influenza A virus (Fig. 1).

**Table 1:** Moving Epidemic Method (MEM) threshold levels for influenza epidemic activity

Threshhold level (× 100.000 h)	baseline	low	medium	high
SHLCI	1.82	9.18	20.91	30.1
SHLCI-FLUA	1.33	7.07	23.36	39.61
SHLCI-FLUB	0.5	1.56	4.27	6.25
PIDIRAC-ILI	74.00	293.45	425.12	500.8
PIDIRAC-FLU	13.35	59.48	84.98	99.5
PIDIRAC-FLUA	10.06	33.99	90.52	139.56
PIDIRAC-FLUB	5.95	24.97	57.66	83.47

*SHLCI* Severe hospitalized laboratory confirmed influenza, *SHLCI-FLUA A* Severe hospitalized laboratory confirmed influença type A, *SHLCI-FLUA B* Severe hospitalized laboratory confirmed influença type B, *PIDIRAC-ILI* Incidence rate for Primary care inflenza like illness, *PIDIRAC-FLU* Incidence rate for Primary care influenza laboratory confirmed cases, *PIDIRAC-FLUA* Incidence rate for Primary care influença type A laboratory confirmed cases, *PIDIRAC-FLUB* Incidence rate for Primary care influenza type B laboratory confirmed cases

**Fig. 2:**
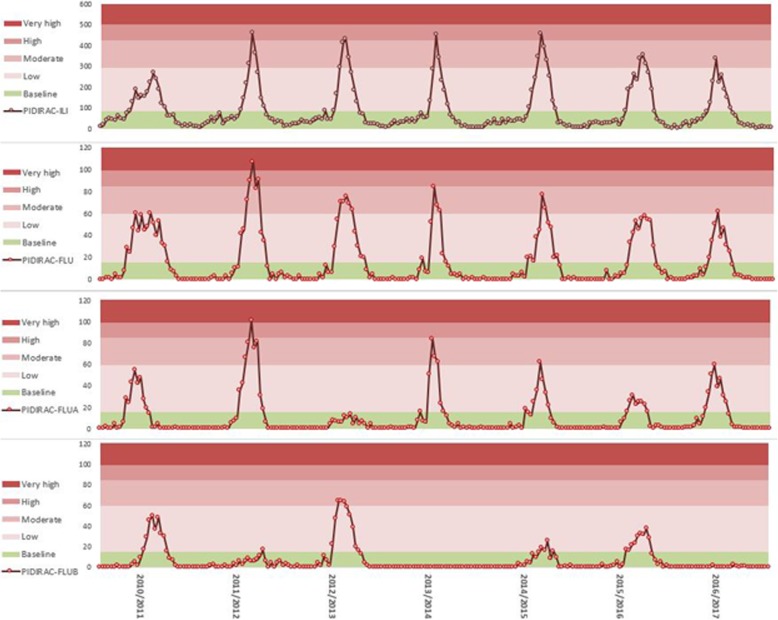
Epidemic curve and MEM levels (baseline, low, moderate, high and very high) estimated for PIDIRAC data source. Influenza epidemic seasons 2010–11 to 2016–17

Epidemic intensity reached moderate threshold levels for ILI incidence rates and influenza virus isolate incidence in all epidemic seasons except for the 2010–2011 post-pandemic season, when the duration of the epidemic was longer in time but of lower intensity. Highest peak incidence levels were reached in 4 seasons from 2011 to 2015. In seasons with co-circulation of both A and B influenza virus, individually assessed intensity did not exceed moderate level (Fig. 2). Influenza A epidemic curves presented higher peaks of epidemic intensity than B influenza virus but with shorter duration.

Assessment of the SHLCI source showed an increase in the rate of cases for the four last seasons (2013–2017) although threshold level reached did not surpass the moderate level and did not reach high intensity level.

**Fig. 3:**
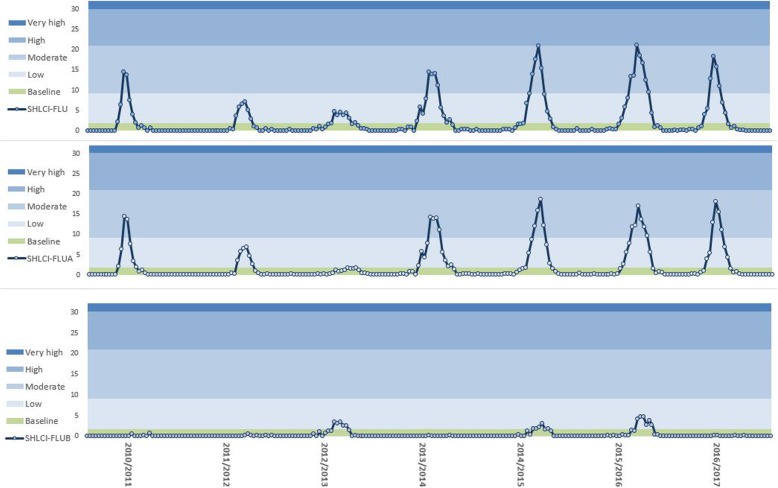
Epidemic curves and MEM levels (Baseline, low, moderate, high, very high) estimated for data source SHLCI Influenza epidemic seasons 2010–11 to 2016–17

Incidence rates of SHLCI-FLUA cases reached peak values of 15–20 cases/100,000 inhabitants, which are set within the moderate intensity level during epidemic weeks, while incidence rates of SHLCI-FLUB were less frequent and displayed lower incidence rates of 3–5 cases/100,000 inhabitants (Fig. 3), slightly exceeding baseline threshold level in those seasons with greater B type virus circulation.

**Fig. 4:**
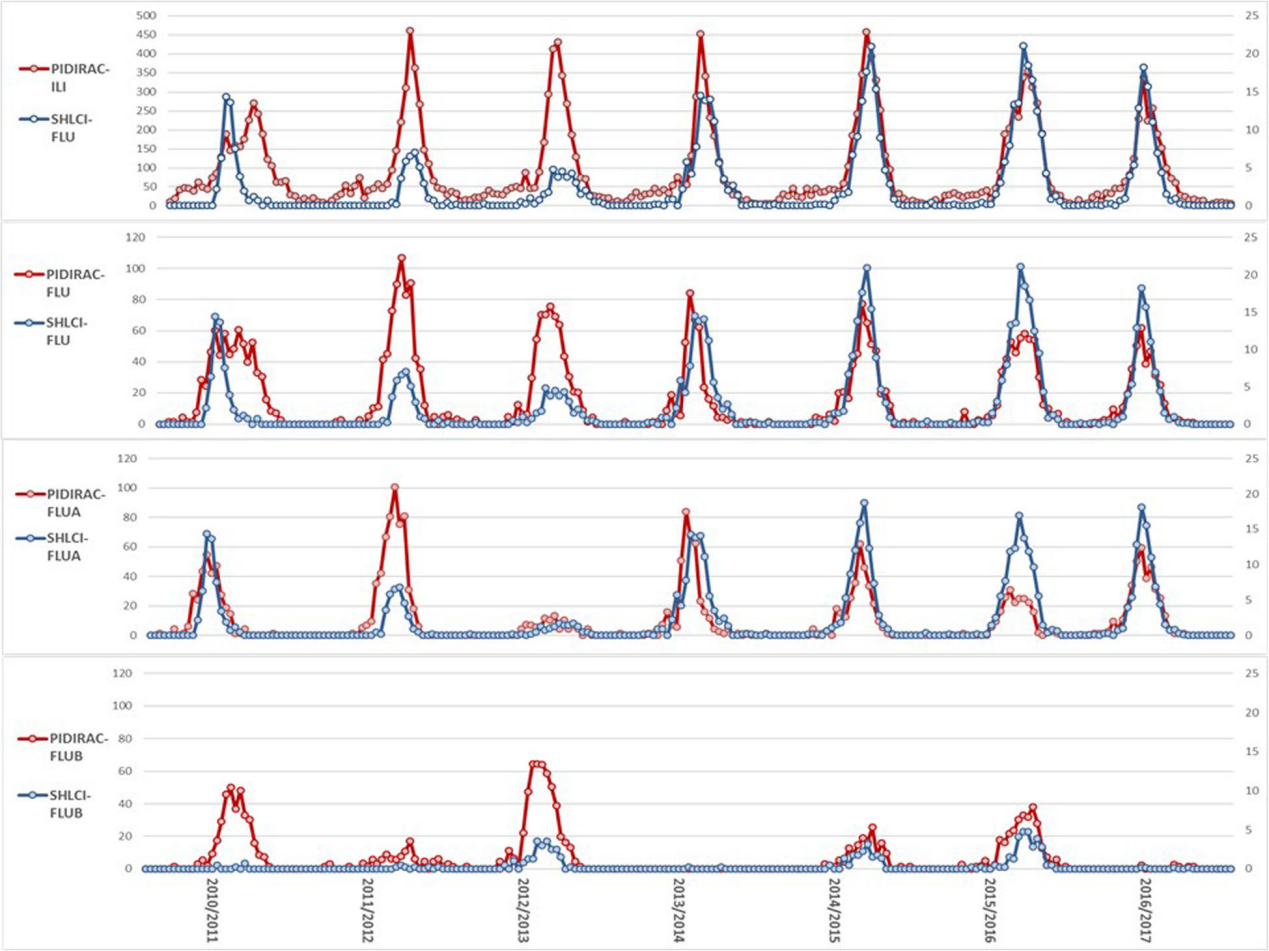
Comparison of both surveillance systems by data source pairs: PIDIRAC and SHLCI

When comparing PIDIRAC–FLU and SHLCI, a decreasing severity trend was observed for the first three seasons, while for the latter four seasons the trend was opposite showing a constant increase in SHLCI incidence with respect to PIDIRAC–FLU (Fig. 4). SHLCI-FLUB cases in seasons 2014/2015 and 2015/2016 showed a marked increase with respect to PIDIRAC-FLUB. Paired incidence curves for SHLCI -FLU and PIDIRAC-ILI and SHLCI -FLUA and PIDIRAC-FLUA showed the best correlation index (0.805 and 0.724 respectively).

Comparison of the curves according to MEM intensity levels, paired curves that showed higher correspondence in their levels of intensity was the SHLCI -FLUB/PIDIRAC-FLUB pair, being above 85% globally and 55% during epidemic weeks (Table 2). Assessing delay in reaching epidemic level, the PIDIRAC-ILI source forecasts an average of 1.6 weeks before SHLCI. Differences are higher when SHLCI cases are paired to PIDIRAC-ILI and PIDIRAC-FLUB (SHLCI-FLU/PIDIRAC-ILI and SHLCI-FLUB/PIDIRAC-FLUB), although statistical significance was observed only for SHLCI-FLU/PIDIRAC-ILI (*p*-value Wilcoxon test = 0.039) (Table 2).

**Table 2:** Delay in achieving epidemic threshold and correlation between curves and MEM epidemic levels

Influenza season	SHLCI / PIDIRAC-ILI	SHLCI / PIDIRAC-FLU	SHLCI -FLUA / PIDIRAC-FLUA	SHLCI -FLUB / PIDIRAC-FLUB
2010_11	1	1	1	na
2011_12	2	2	2	na
2012_13	6	3	na	3
2013_14	1	1	1	na
2014_15	1	2	2	1
2015_16	0	-1	-1	4
2016_17	0	0	0	na
mean ± SD	1.6 ± 2,1	1.1 ± 1.3	0.8 ± 1.2	2.7 ± 1.5
*P*-value	0.039	0.071	0.129	0.109
Correlation (LN)	0.805 (0.814)	0.694 (0.749)	0.724 (0.743)	0.583 (0.666)
MEM correlation	0.745	0.649	0.752	0.689
Global ILSR	81.0%	77.5%	80.8%	85.9%
Epidemic ILSR	47.4%	38.2%	46.0%	58.8%

*Na* not available, *LN* natural logarithm, *ILSR* Influenza Likelihood Signed Rank, *MEM* Moving Epidemics Method, *SHLCI* Severe hospitalized laboratory confirmed influenza, *SHLCI-FLUA A* Severe hospitalized laboratory confirmed influença type A, *SHLCI-FLUA B* Severe hospitalized laboratory confirmed influença type B, *PIDIRAC-ILI* Incidence rate for Primary care inflenza like illness, *PIDIRAC-FLU* Incidence rate for Primary care influenza laboratory confirmed cases, *PIDIRAC-FLUA* Incidence rate for Primary care influença type A laboratory confirmed cases, *PIDIRAC-FLUB* Incidence rate for Primary care influenza type B laboratory confirmed cases

## Discussion

This study is based on surveillance data of ILI incidence rates from primary care sentinel surveillance, virology results from sampling by sentinel primary care physicians and severe laboratory-confirmed influenza (SHLCI) that required hospital admission during seven influenza epidemic seasons, from 2010 to 2017, in Catalonia. Data obtained from these seven epidemic seasons showed a good correlation between the two systems, primary care and SHLCI, although some differences in prompt detection of epidemic onset were observed according to predominant circulating influenza virus.

Case definitions with a lower relative specificity for influenza, such as ILI can decrease the specificity of the model to detect an influenza season [16]. This occurs because of the increasing circulation of other respiratory pathogens, such as respiratory syncytial virus that add up to ILI Incidence rates in the primary care system, yet this is not so in the SHLCI because these are all laboratory confirmed influenza cases [16, 17]. Other factors influencing outpatient consultation rates, such as public concern, or the circulation of a novel recombinant influenza virus with pandemic potential, can also act as amplifiers of ILI consultation rates and disease burden results [18]. This may result in patterns with bimodal waves as observed in some seasons such as the 2014–2015 or pre-epidemic peaks as observed in the 2010–2011 season. Nevertheless increase in ILI rates was consistent with increases in influenza laboratory confirmation of primary care sentinel samples as well as severe confirmed influenza hospitalization rates, a fact that has also been observed by other researchers as well [19]. The complementary virological data available in the SHLCI should be more accurate to confirm the start of the epidemic period. In the present study this fact was observed only when there is Influenza B predominant circulation. Although the low incidence of influenza B virus in the seasons studied does not allow to conclude about the benefit of combining the two systems of surveillance respect the surveillance made by PIDIRAC outpatient sentinel System. This fact differs from the observations from Murray et al. that found timing to be the same in all influenza seasons where influenza A (H3N2) was the most prevalent subtype and that using historical data from all influenza seasons regardless of circulating subtype, weeks predicted to be above the epidemic threshold do not change. Meaning that the MEM approach could be useful for planning regardless influenza virus subtype [19].

The present study is an observational study with strengths and limitations. As a strength we can highlight that there are few studies investigating timeliness of sentinel primary care vs SHLCI to predict the onset of the epidemic allowing for on time preparedness to cope with the influenza epidemic and will allow for timely assessment of intensity and severity of an influenza epidemic.

This study reveals the importance of both systems to understand yearly influenza epidemic behavior and to strengthen healthcare resource preparedness.

Although MEM was primarily developed for modeling ILI data, it has been proven successful with SHLCI data showing that influenza severe cases’ waves correlate well with ILI, with higher epidemic thresholds and shorter range among intensity thresholds. Intensity, understood as the level of the population consultation rates or the percentage of ILI primary care consultations which is not synonymous of intensity or severity specific to influenza, but has proven to be one of the most reliable indicators to describe the impact of influenza on the population. On the other hand, SHLCI can complement primary care indicators giving a picture of the severity of the epidemic. Comparing intensities and severity across seasons is essential for the understanding of seasonal epidemic patterns and of future pandemics, and to assess control measures, such as the effectiveness of vaccination campaigns [20].

As a limitation we consider the fact that a certain proportion of the population will not seek medical attention. Other complementary systems could possibly account for those who do not seek health care attention for ILI have been described such as Influweb or internet flu consultation trends such as Google flu trends, although these systems have, by themselves alone, low representability [3, 21].

An epidemic threshold was determined for the ILI consultation rates reported by the sentinel physicians and for the SHLCI to assess the period of increased influenza epidemic earlier. Yet on time complete reporting of hospitalized severe cases is, at the best scenario, on a week’s delay, making this system unable to predict an overload in hospital resource demand with anticipation. Nevertheless the surveillance of SHLCI has the potential to reveal timely insights into epidemic severity as has been also pointed out by other authors [22–24].

Besides, the voluntary participation of hospitals that could account for a selection bias. However, although a selection bias cannot be ruled out, the 12 participating tertiary hospitals cover 62% of population of Catalonia meaning that the effect can be assumed not to invalidate data.

Another limitation is the representativeness of the samples tested in primary care by the systematic sampling of two first weekly ILI patients attended. Other circulating respiratory virus can mimic influenza and contribute to increase in ILI incidence rates, not so in SHLCI that are all influenza confirmed. Thus, increase in hospitalization of SHLCI reflects true influenza virus circulation, albeit a certain delay depending on circulating virus predominance. The implementation of a third hospital warning system based on data from emergency department records for laboratory confirmed influenza would be desirable to further improve timely information to health care for appropriate response.

Another limitation to determining burden of hospitalization due to confirmed influenza is presented by the lack of the inclusion in the sentinel surveillance system of a data source for Influenza hospitalization. The use of other data systems such as hospital discharge data might spare the underestimation of the burden of laboratory confirmed influenza hospitalization [25]. Improvement to upgrade the assessment of influenza burden could be achieved by adding an automated surveillance system based on ICD − 10 codes assigned by physicians after emergency room consultation [26].

## Conclusions

Results of two different surveillance systems during seven influenza seasons studied showed similar trends and were highly correlated with each other. The ILI consultation rates reported by the sentinel primary care physicians remain the basis of surveillance in Catalonia, because the system integrates epidemiological and virological information. Hospital data and virological data will remain very important to provide information on the severity, molecular and phenotypic characterization of the viruses, and whether the ILI incidence is truly related to influenza virus infections.

The combined PIDIRAC Influenza sentinel surveillance system provides timely and accurate syndromic and virological surveillance of influenza from the community level to hospitalization of severe cases. The implementation of a hospital warning system based on data from emergency department records would be desirable as a complementary component of the combined outpatient-SHLCI scheme, allowing to inform control measures to lessen the burden of influenza in Catalonia and ensure access to quality timely information to health care for accurate preparedness.

## Data Availability

Data is available upon request by the corresponding author of the manuscript.

## Abbreviations

CI: Confidence interval
FLU: Laboratory confirmed influenza
FLU-A: Laboratory confirmed influenza A virus
FLU-B: Laboratory confirmed influenza B virus
ILI: Influenza like illness
MEM: Moving Epidemics Method
PIDIRAC: Daily Acute Respiratory Infection Sentinel Surveillance System of Catalonia
SHLCI: Severe Hospitalized Laboratory Confirmed Influenza
